# Effects of Marine Bioactive Compounds on Gut Ecology Based on In Vitro Digestion and Colonic Fermentation Models

**DOI:** 10.3390/nu14163307

**Published:** 2022-08-12

**Authors:** Min Wang, Jianjun Zhou, Joaquim Calvo-Lerma, Yixuan Liu, María Carmen Collado, Francisco J. Barba

**Affiliations:** 1 Preventive Medicine and Public Health, Food Science, Toxicology and Forensic Medicine Department, Faculty of Pharmacy, Universitat de València, Avda. Vicent Andrés Estellés, 46100 Burjassot, València, Spain; 2 Institute of Agrochemistry and Food Technology, National Research Council (IATA-CSIC), 46980 Paterna, València, Spain; 3 Key Laboratory for Deep Processing of Major Grain and Oil, Wuhan Polytechnic University, Wuhan 430023, China

**Keywords:** in vitro digestion, colonic fermentation, marine bioactive compounds, digestion model

## Abstract

Digestion and the absorption of food compounds are necessary steps before nutrients can exert a role in human health. The absorption and utilization of nutrients in the diet is an extremely complex dynamic process. Accurately grasping the digestion and absorption mechanisms of different nutrients or bioactive compounds can provide a better understanding regarding the relationship between health and nutrition. Several in vitro models for simulating human gastrointestinal digestion and colonic fermentation have been established to obtain more accurate data for further understanding of the metabolism of dietary components. Marine media is rich in a wide variety of nutrients that are essential for humans and is gaining increased attention as a research topic. This review summarizes some of the most explored in vitro digestion and colonic fermentation models. It also summarizes the research progress on the digestion and absorption of nutrients and bioactive compounds from marine substrates when subjected to these in vitro models. Additionally, an overview of the changes imparted by the digestion process on these bioactive compounds is provided, in order to support those marine resources that can be utilized for developing new healthy foods.

## 1. Introduction

Despite the characterization of food composition in nutrients and bioactive compounds being well established, the process of human gastrointestinal digestion consists of a series of changes determining the eventual fate and function of food components. Therefore, the follow-up of any food component of interest should be specifically addressed depending on the digestion context and the food matrix [1,2], as both the structure and composition of food have an impact on the digestion process [3]. Throughout digestion, complex food components are released from the food matrix and are broken down into simpler structures—mostly single-component substances—which are then absorbed. When absorbed at specific locations, nutrients exert their physiological function. Therefore, it is necessary to understand the release mechanisms and the extent of the absorption of food components during digestion [4].

The human digestive system is mainly composed of the digestive tract, including the mouth, stomach, small intestine, large intestine, and other assisting organs [5]. When food enters the oral cavity, it is broken down into small parts by the mechanical action of the teeth and, simultaneously, an oral bolus is formed by mixing with saliva. The enzymes contained in saliva (amylases) then begin the breakdown of starch. During this step, oral microbes are actively involved. Oral microbes can break down the food structure, thus facilitating the digestion process and playing a role like that of enzymes. In addition, oral microbes persist in the gut, which are closely related to gastrointestinal health [6]. The action of swallowing pushes the food (plus the oral mixture) to the stomach through the esophagus. From there, hydrolysis of protein begins via the action of gastric juice, which contains pepsin. The contraction of the stomach wall muscles continually disrupts the matrix, contributing to reductions in the particle size of the food. After 1–3 h, and once the particle size is small enough to pass through the pylorus, the gastric content enters the small intestine. The small intestine is composed of the duodenum, jejunum, and ileum, where most food components are absorbed. Pancreatic secretions and bile are delivered into the duodenum to participate in digestion. Pancreatic secretions contain trypsin, pancreatic lipase, pancreatic amylase, and other enzymes, which can hydrolyze various components including lipids, proteins and carbohydrates into smaller molecules. Bile contains bile salts which participate in the digestion and absorption of lipids. In addition, the lining of the small intestine has a special brush border structure which is the final site of food digestion. Digestive enzymes secreted by the small intestinal epithelial cells can hydrolyze peptides or disaccharides into amino acids, glucose and other substances that can be readily absorbed by the intestinal wall and circulate within the body’s fluids [7,8]. The large intestine can absorb water, while the solid fraction, including un-digested or un-absorbed matter, is excreted. The large intestine is an anaerobic environment, with an abundance of microorganisms considerably higher than that present in the small intestine and stomach. Among them, the number of anaerobic bacteria is also much higher than that of aerobic bacteria, and the enzymes produced by these microorganisms can hydrolyze food components that the upper digestive tract cannot, such as dietary fiber [9]. This way the gut microbiota can obtain nutrients from the diet and maintain a synergistic relationship with the host.

Among the biological systems that are considered as foods, different origins contribute to the composition of nutrients, imparted by environmental constituents. In this sense the ocean is a huge treasure trove of resources and an important part of the biodiversity of ecosystems. The rational use of marine resources can help to alleviate food pressure caused by population growth. About half of the available marine organisms such as fish, shellfish or macroalgae can be directly used for food consumption after harvesting. In particular, microalgae can also be consumed in other forms [10]. These marine organisms contain relevant nutrients that can serve as an important source of bioactive compounds, including polyunsaturated fatty acids (PUFAs), polysaccharides, polyphenols, and protein among others. Microalgae components have great potential applications, and exhibit multiple positive health-related benefits, such as anticancer, anti-diabetic and antibacterial properties [11,12]. However, a large amount of waste can be generated in the process of aquatic product processing. For example, fish heads and viscera are discarded as waste during the processing of fish, and for crustaceans, only about 10% of the organism can be eaten. These discarded pieces or parts are rich in bioactive compounds that results in a loss and a waste of resources [13]. For this reason, in recent years, bioactive compounds from the ocean—some of them generated as by-products—have begun to attract attention. For example, collagen from fish bones and chitosan from shrimp shells have been shown to promote the proliferation of lymphocytes without damaging cells, and PUFAs from fish visceral and microalgae have also been found to have anti-inflammatory properties [14,15]. This review aims to provide an overview of the digestion process of various bioactive compounds from marine organisms and their potential impact on host health, according to research findings based on in vitro digestion and colonic fermentation models.

## 2. In Vitro Gastrointestinal Digestion Models

Food digestion is a complex, continuously changing dynamic process that is affected by many factors. Understanding the physical (food matrix degradation due to mechanical forces such as peristalsis) and chemical (enzymatic activity breaking down macromolecules) changes in food during digestion enables the rational evaluation of nutrient release and bioaccessibility and provides a theoretical basis for designing a balanced diet and improving health [16]. Considering the limitations of using humans and animals as exploratory models, increased attention has been paid to in vitro digestion models, and a variety of in vitro digestion models from static and semi-dynamic to dynamic have been developed. Depending on the purpose of the study, digestion models are designed with the pertinent specifications, involving the stages of oral, gastric, and small intestine, and in some cases colonic (large intestine) fermentation is also included [17].

### 2.1. Static Digestion Models

**Figure 1:**
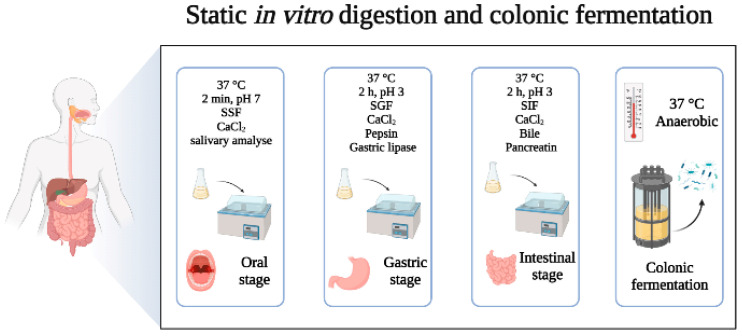
Flow diagram ofstatic in vitro digestion and colonic fermentation models. SSF: simulated salivary fluid; SGF: simulated gastric fluid; SIF: simulated intestinal fluid. Adapted from Brodkorb et al. [19] and Carbonell-Capella et al. [24].

Many in vitro digestion models are designed to simulate digestive fluids, peristalsis of the human gastrointestinal tract with agitation under static conditions, and to achieve temperature and pH adjustment through equipment such as water baths, shakers, and pH-meters. In 2014, the conditions and procedures for static digestion models were standardized in the framework of the COST action INFOGEST into an international and harmonized protocol, and thereafter updated in 2019 (Figure 1). The digestion process was defined in terms of the key parameters and experimental conditions that should be common in all studies [18,19] and which should be adapted to the purpose of the study. The static models are useful for screening purposes or as a first step in understanding the digestion of a compound, but present with several underestimations and limitations of the physiological process. These models have been used to simulate the digestion process of some purified compounds from foods, such as protein hydrolysis, starch resistance, etc., and they can preliminarily explore the rationality of nutrient digestion, with the advantages of fast and easy operation [20,21]. In addition, progress in understanding nutrient digestion in complex food matrices has been achieved by following the static INFOGEST protocol [22]. However, in the process of food digestion, the dynamic incorporation of the digestive fluids at specific volumetric flow rates, the dynamic passage of digesta from one compartment to another, and the grinding force of digestive organs are also important factors. The static digestion models only simulate the process through mechanical and continuous stirring, which oversimplifies the changes in the physiological environment. Therefore, static models cannot accurately simulate the dynamic changes in the whole process, entailing several limitations [23]. 

### 2.2. Dynamic Digestion Models

In order to overcome the limitations of static digestion models, researchers have developed a variety of dynamic in vitro digestion models ranging from single-compartment to multi-compartment systems which can better simulate the behavior of food in the digestive system. 

#### 2.2.1. Mono-Compartment System

- Dynamic gastric model (DGM)

The dynamic gastric model is a computer-controlled, mono-compartment system developed by the Institute of Food Research (Norwich, UK) to simulate the mechanical effects of human gastric digestion. The model mainly consists of two parts: fundus/main body (proximal stomach) and antrum (distal stomach), whereby enzyme and acid solution are added through the fundus portion and dynamically delivered by a computer-controlled pump, distributed outside the body of the DGM [25]. In the DGM cavity, there are movable barrels and pistons. The shearing force they generate when they move can reduce the particle size of the food, while simultaneously simulating the digestion and decomposition of the stomach and the emptying of food in real time [26]. However, the main body and gastric antrum are vertically distributed in this model, which is different from the actual digestive system.

- Human gastric simulator (HGS)

The human gastric simulator (HGS) is mainly used to simulate the peristaltic activity of the lower part of the stomach (gastric antrum), which is composed of a cylindrical latex gastric cavity, a mechanical drive device, a reservoir, a secretion and a temperature control device. The entire HGS unit is installed in a foam plastic chamber with heaters and fans, allowing for operation at 37 °C [27]. The advantage of HGS is the generation of mechanical force and the ability to adjust indicators such as the gastric secretion rate according to different physiological conditions, in order to achieve the same effect as in vivo digestion. However, this model has limitations as it cannot simulate the true shape of the stomach, and at the same time it also does not include the oral and small intestine stages [28].

#### 2.2.2. Multi-Compartment System

- In vitro dynamic system (DIDGI)

DIDGI is a system developed by the French National Institute for Agricultural Research (INRA, Rennes, France) to simulate the digestive process, namely the two consecutive compartments of the stomach and small intestine with two glass jackets filled with water drawn from a temperature-controlled water bath. The sieving function of the pylorus is mimicked by a Teflon membrane between the gastric and intestinal compartments [29]. The system uses a computer to control and monitor the transport of food in the stomach and intestines, including food transport, pH changes, digestive secretions, emptying rates, etc. [30]. Compared with HGS, DIDGI is transparent, so process-induced changes in the food form during digestion can be visually monitored. In contrast, it cannot simulate the digestive process of the small intestine stage [31].

- TNO gastrointestinal model (TIM)

The in vitro gastrointestinal model (TIM) is a computer-controlled multi-compartment dynamic system developed in 1992 at the TNO Nutrition and Food Research Center (Netherlands) [26]. The model consists of systems such as TIM-1, TIM-2, Tiny TIM and TIM-agc. The TIM-1 system has four compartments, including the stomach, duodenum, jejunum and ileum, which simulate the continuous dynamic environment of the upper gastrointestinal tract and is the most commonly used model [32]. The TIM-2 system can simulate the environment of the colon, allowing for study of the metabolic action of the gut microbiota [33]. Tiny TIM contains the stomach component of TIM-1, as well as the small intestine part which is only one compartment and is more simplified compared to the TIM-1 system. The TIM-agc model is a more advanced gastric model that simulates behaviors such as peristaltic contractions of the stomach and the gastric antrum [34]. For the TIM system temperature, pH, peristalsis, and transport flexibility can be controlled with high precision by computer software. It can process different food components according to research purposes and simulates different states of the gastrointestinal tract environment, rendering it a reliable in vitro digestion model [35].

- Simulator of the human intestinal microbial ecosystem (SHIME^®^)

The simulator of the human intestinal microbial ecosystem (SHIME) model was developed by Molly et al. in 1993 [36] and has been continuously improved. The model consists of five sensors that work together to simulate the digestive process from the mouth to the large intestine. Meanwhile, it also combines with gut microbes, enabling the ability to obtain relevant information related to human health by assessing changes in microbial composition and metabolism through long-duration experiments [37]. Following refinement of the model, M-SHIME and TWIN-SHIME were developed to simulate in parallel the digestive conditions associated with various diseases, such as inflammatory bowel diseases [38,39], at the time the experiment was run at the standard conditions.

- Engineered stomach and small intestinal system (ESIN)

To overcome the shortcoming of models such as TIM and SHIME, an engineered stomach and small intestine model (ESIN) was developed in 2012 (University of Auvergne, France). ESIN is a multi-compartment system consisting of six compartments, including the meal reservoir (food storage), saliva ampoule, stomach, duodenum, jejunum and ileum [40]. The gastric compartment is set up in a cylindrical compartment, and food that is representative of the actual size of a real diet enters the stomach through the meal reservoir. Particles between 1~2 mm in size and liquids can pass through the pylorus, and larger particles continue to be further digested in the stomach [41]. Meanwhile, both open ends are connected to a peristaltic pump programmed to simulate different gastric emptying scenarios for liquids and solids [42]. As a multi-compartment model, ESIN can be used to simulate the digestive behavior of food with a size similar to regular meals.

In addition to these earlier designed and used models, many others have also been developed to simulate in vitro digestion. These include the dynamic gastric simulating model (DGSM) developed at the University of Leeds (Leeds, UK) to assess the role of enzymes in food digestion [43]; a 3D printed in vitro mechanical gastric system (IMGS) constructed with the flexible gastric compartment, which can evaluate the real gastric peristalsis behavior [44]; and the near real dynamic in vitro human stomach system (new DIVHS) designed by Soochow University (Suzhou, China) where part of the stomach is also produced by 3D printing technology, which can impart the adjustment of gastric emptying rate [45]. In addition, there are also models that can simulate the digestive system of infants and, specifically, are developed to assess the digestibility of infant food including breast milk [46]. 

## 3. In Vitro Colonic Fermentation

In recent years, through these aforementioned digestion models, researchers have explored the bioaccessibility of the various bioactive components of food, namely the portion of a compound that is released from the food matrix in the gastrointestinal tract that can be absorbed. In this sense, the generated knowledge has helped to predict or estimate the in vivo effects of different food components, and to develop new foods that are healthy and nutritious [17]. It is worth noting that SHIME, TIM and SIMGI also simulate the behavior of the colon stage, taking into account the influence of the gut microbes [47].

There are about one trillion microorganisms existing in the human gut, referred to as the gut microbiota [48,49]. In recent years, the importance of the gut microbiota has become well known, as it influences health through the action of their metabolites and their derivatives [50,51]. For instance, there exists a close interaction between the gut microbiota ecosystem and the immune system, which plays a key role in human physiology and metabolism. Microbiota metabolites can exert a regulatory effect on the central nervous system of the human, therefore affecting behavior [52,53]. 

Anaerobic fermentation of non-digested or non-absorbed food components is one of the main functions of the colon. During the fermentation process, some oligosaccharides that escape gastrointestinal digestion are used as substrates by the microorganisms, and short-chain fatty acids (SCFAs) are produced because of metabolism. Fermentation also leads to the production of gases and other metabolites [54,55]. Although the fermentation process occurs in the large intestine, it can affect the metabolism and immunity of the human body through the transmission of signal molecules that are absorbed and pass to the bloodstream, such as SCFAs. Therefore, a suitable in vitro fermentation model is an effective tool to study the effects of different food components on the growth of gut microbiota and the production of metabolites.

### 3.1. In Vitro Colonic Fermentation Models

In vitro colonic fermentation is conducted by cultivating isolated gut microbiota. These are derived from fecal samples or bacterial consortiums at a specific pH, temperature, substrate concentration and time, under anaerobic conditions, to explore the effect of the substrate on the growth and metabolism of the gut microbiota. As with in vitro digestion models, in vitro fermentation models have evolved from simple static models in the beginning to dynamic models that can evaluate different biological components. At present, the in vitro colonic fermentation models can be roughly divided into two categories: static batch culture and dynamic continuous culture.

#### 3.1.1. In Vitro Static Batch Fermentation Models

Static batch fermentation models are used to reproduce fermentation in several samples simultaneously with screening purposes. In these models, single strains or isolates from fecal samples are added to a certain amount of culture medium and the compound of interest is then incorporated. The mixture is placed in a closed anaerobic fermenter, or a sealed tube preserved at 37 °C [56]. In order to more accurately simulate colonic fermentation, the compound of interest should have undergone gastrointestinal digestion prior to fermentation. The static batch fermentation model uses single or multiple fecal samples as inoculum, and the simulation is reproduced for at least 24 h (normally up to 48, as thereafter accumulation of metabolism products disrupts a representative situation) and no new substrate is added during the process. These models are mostly used to study the effects of substrate on the physiology and metabolism of gut microbiota, such as the differences between doses and sources of substrates [57,58]. At the same time, the metabolic processes of gut microbiota, such as the production of short chain fatty acids, can be explored through static batch fermentation models. This allows for a more comprehensive assessment of the interactions between substrates and gut microbiota, with the advantage of being simple and fast [59]. However, these models have shortcomings because the entire fermentation environment is sealed, there is no substrate or medium supplementation, or there is consumption of nutrients and accumulation of metabolites during the process, which can alter the growth of gut microbiota [60]. Another relevant limitation of these models is the time duration, as it only allows for evaluation of the 48 h effect of the studied substrates, while most of the food components considered as prebiotics account for significant changes in the gut microbiota after an exposure time of weeks.

#### 3.1.2. In Vitro Dynamic Continuous Models

In order to overcome the substrate consumption and metabolite accumulation problems of static batch culture and the duration of the experiment, some dynamic models have been developed in order to establish in vitro colonic fermentation systems [61]. These continuous culture models can be used to assess the impact of dietary components on the gut microbiota, which is closer to the real human environment. A variety of dynamic fermentation models have been developed, including the TIM, SHIME and SIMGI mentioned above. This is in addition to some models that are dedicated to colonic fermentation, such as the Artificial Colon (ARCOL) system which can simulate the main parameters of human colonic anaerobic fermentation, including temperature, retention time, water and metabolite absorption. In this system, a mixture of protein, carbohydrates and other nutrients are used as the ileal effluent and nitrogen is used to maintain an anaerobic environment. Meanwhile, a hollow cellulose dialysis membrane is used to maintain the concentration of electrolytes and metabolites [62]. Most of these dynamic models are developed based on the model of Gibson et al. and consist of three vessels with the appropriate pH to simulate the proximal, transverse and distal colon [63]. In the dynamic model, various factors including the pH, temperature and gaseous environment can be controlled to maintain the normal metabolism of gut microbiota. In general, in vitro static batch fermentation models are used more widely. In Table 1, the advantages and disadvantages of various in vitro digestion and fermentation models are summarized.

## 4. In Vitro Digestion and Fermentation of Marine Bioactive Compounds

### 4.1. Polysaccharides

Polysaccharides exist widely in various organisms and play an important part in the human diet. They differ from carbohydrates such as starch, as most polysaccharides from marine organisms have a different composition and structure from terrestrial plants; they can act as prebiotics by resisting the action of digestive enzymes in the stomach and small intestines [64]. Some studies have shown that the digestion process of polysaccharides is diverse (Table 2). For example, the *Gracilaria* polysaccharide undergoes no release of monosaccharides nor changes in molecular weight during gastrointestinal digestion, indicating that it is not absorbed by the digestive system. Whereas sea cucumber polysaccharide changes its molecular weight during the digestion process [65,66,67]. Marine polysaccharides that escape intestinal digestion enter the colon, where they are utilized as substrates by microbes. The gut microbiota has the function of encoding carbohydrate-active enzymes (CAZymes), which can further degrade indigestible marine polysaccharides into monosaccharides or other metabolites, with the potential to have an impact on the host’s health [68]. 

**Figure 2:**
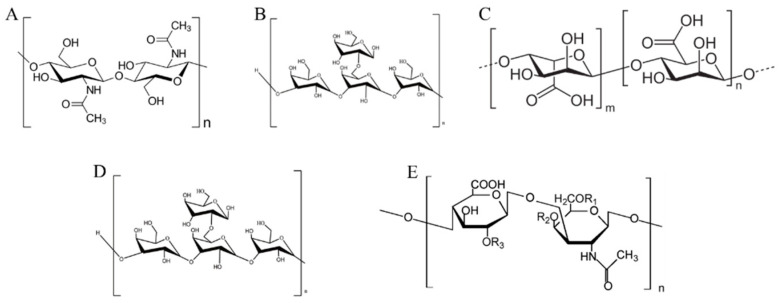
Polysaccharides from marine organisms: (**A**) chitin, (**B**) laminarin, (**C**) alginate, (**D**) fucoidan, (**E**) chondroitin sulfate.

Marine polysaccharides exist in animals (chitin and laminarin), algae (alginate and fucoidan) and various microorganisms. These polysaccharides have the potential to improve intestinal health and relieve intestinal inflammation and various other diseases [69], so their digestion and absorption has also been a relevant focus for study (Figure 2). Algae have attracted the attention of researchers due to their wide variety and high polysaccharide content. Di et al. [70] established an oral-gastric small intestine model to simulate the digestion behavior of sulfated polysaccharides from *Gracilaria rubra*. The analytical determinations of reducing sugars and molecular weight showed that these polysaccharides were not digested. Subsequent in vitro fermentation showed that these polysaccharides could be utilized by gut microbiota to produce more SCFAs, including acetate and propionate. Additionally, sulfated polysaccharides from *Gracilaria rubra* decrease the *Firmicutes*/*Bacteroidetes* ratio, thus showing potential for development as a prebiotic. Chen et al. [71] explored the in vitro digestibility and fermentation capacity of *Ascophyllum nodosum* polysaccharides. In vitro simulation of oral and gastrointestinal tract digestion showed that amylase, gastric juice and intestinal juice had no effect on the polysaccharides, but the molecular weight of the polysaccharides-reducing sugars decreased significantly after fermentation by the gut microbiota. It also increased the abundance of *Bacteroidetes* and *Firmicutes*, contributing to the production of SCFAs. Further, Wang et al. [72] obtained four different purified fractions of polysaccharides from *Coralline pilulifera* through separation and purification, as well as explored the most important fraction through the in vitro digestion and fermentation system. By measuring the molecular weight before and after digestion, it was shown that this fraction could not be digested. Through in vitro fecal fermentation, similar results as in the previous studies were found. The gut microbiota could decompose undigested polysaccharides, reducing their content by about 70% within 24 h. This promoted the growth of beneficial bacteria and SCFAs content. Slightly different from this study, Li and co-workers [73] confirmed via an in vitro digestion model that *Nostoc commune* Vauch polysaccharides were degraded during digestion, contributing to the improvement of antioxidant capacity. These have also been shown to have a positive effect on regulating gut microbiota and SCFAs production. In addition, the digestion and fermentation of various polysaccharides from algae, such as carrageenan, *Silvetia compressa* polysaccharides, etc. [74,75], have been investigated, and many of these polysaccharides have shown positive effects in improving gut microbiota.

In addition to algae, fish and crustaceans in the ocean are also the main carriers of polysaccharides. For example, the polysaccharide content in the exoskeleton structure of shrimp is about 15~40%, and chondroitin sulfate can be isolated from fish cartilage [76,77]. These polysaccharides have also proven to have a positive effect on human health. For example, Pacific abalone has received attention as a high-value economic crop. Ai et al. [68] explored the absorption and degradation mechanism of sulfated polysaccharides in abalone by establishing in vitro and in vivo models. The digestion process of purified abalone polysaccharide was simulated by an in vitro model, and the molecular weight of sulfated polysaccharide did not change before and after digestion, indicating that the simulated digestive juice had no effect on the polysaccharide. Further analysis of the metabolites after colonic fermentation revealed an effect of this polysaccharide on the gut microbiota and an exhibition of bioactivity. On the other hand, a static in vitro digestion model was used to evaluate the digestion process of fucosylated glycosaminoglycan obtained from sea cucumber [66]. After saliva-gastric intestinal digestion, there was no release of free monosaccharides. At the same time, it proved to inhibit various digestive enzymes including pepsin, so its potential to prevent obesity and other diseases has been suggested. This study only explored the digestive process from the oral cavity to the small intestine. Another study explored the effect of sea cucumber polysaccharides on the gut microbiota through an in vitro static batch colonic fermentation model, and found that the consumption of polysaccharides during fermentation contributed to the accumulation of beneficial microbial metabolites [78]. Some crustaceans are also important sources of marine polysaccharides. Ma et al. [79] assessed the effect of the *Crassostrea gigas* polysaccharides on gut microbiota using an in vitro simulated digestion and fermentation model. The molecular weight of the *Crassostrea gigas* polysaccharides decreased and the content of reducing sugars increased during the fermentation process, indicating that some polysaccharides were degraded.

**Table 2:** In vitro digestion and colonic fermentation of marine polysaccharides.

Source	In Vitro Digestion Stages and Model	Colonic Fermentation	Results	Ref.
*Gracilaria chouae* sulfated polysaccharides	Static digestion model	Static batch fermentation	Polysaccharides are slightly degraded; different extraction methods have an effect on the enteric fermentation of polysaccharides.	[65]
Sea cucumber fucosylated glycosaminoglycan	Static digestion model		There is no release of free monosaccharides.	[66]
Abalone *sulfated polysaccharides*	TIM model	Mice model/Static batch fermentation	Simulated digestive juices have no effect on polysaccharides, which regulate gut microbiota.	[68]
*Gracilaria rubra* sulfated polysaccharides	Static digestion model	Static batch fermentation	Polysaccharide is not digested by gastrointestinal tract;fermentation produces acetic acid, propionic acid, etc., and reduces the ratio of *Firmicutes*/*Bacteroidetes*.	[70]
*Ascophyllum nodosum* polysaccharides	Static digestion model	Static batch fermentation	Polysaccharide is not digested by saliva and gastrointestinal tract;colonic fermentation reduces the molecular weight of polysaccharides and reduces sugars;increases the relative abundance of *Bacteroidetes* and *Firmicutes*.	[71]
*Coralline pilulifera* polysaccharides	Static digestion model	Static batch fermentation	Polysaccharide is not digested by gastrointestinal tract;after 24 h of in vitro fermentation, polysaccharide content is reduced by 70%.	[72]
*Nostoc commune* Vauch polysaccharides (NCVPs)	Static digestion model	Static batch fermentation	Degradation of polysaccharides occurs during digestion process;NCVPs have the potential to promote intestinal metabolism.	[73]
κ-carrageenans	Static digestion model	Static batch fermentation	κ-carrageenan oligosaccharide was obtained after simulated gastric digestion;κ-carrageenan oligosaccharides with large degree of polymerization enhance the production of SCFAs and increase the abundance of *Bifidobacteria* and *Lactobacillius*.	[74]
Sea cucumber polysaccharides		Static batch fermentation	Fermentation contributes to the accumulation of beneficial microbial metabolites, including propionic acid, butyric acid, amino acid and derivatives.	[78]
Oyster polysaccharides	Static digestion model	Static batch fermentation	A part of the polysaccharides is degraded during the digestion process;indigestible polysaccharides are utilized by the gut microbiota to contribute to SCFAs generation.	[79]
*Gracilaria Lemaneiformis* sulfated polysaccharide	Static digestion model	Static batch fermentation	Sulfated polysaccharide is degraded during fermentation;gut microbes are able to utilize sulfated polysaccharide and produce SCFAs.	[80]
*Laminaria digitata* polysaccharides	Static digestion model	Dynamic continuous models	*Laminaria digitata* polysaccharides resist degradation by digestive enzymes and are fermented by gut microbiota, changing the abundance of *Streptococcus*, *Ruminococcus*, etc. They also increase the concentration of SCFAs such as acetic acid and propionic acid.	[81]

Because only a few marine polysaccharides can be degraded by the digestive tract, some researchers choose to directly use in vitro colonic fermentation models to explore the relationship between polysaccharides and gut microbiota, skipping the simulation of the upper gastrointestinal tract [82]. For example, the in vitro batch fermentation model was used directly to explore the effect of alginate on human gut microbiota. Further analysis of the genome of gut microbiota found that alginate can be degraded by microorganisms. The amount of *Bacteroidetes* and the production of SCFAs increased, showing similar results to the multiple studies described above. Currently, most studies on in vitro digestion and colonic fermentation of marine polysaccharides rely on static models, whereas dynamic models are rarely used. 

### 4.2. Protein

As an important part of the human diet, proteins participate in various physiological functions of the human body and provide energy. Digestion of protein begins in the stomach, and the low pH of gastric juice denatures the tertiary structure of the protein and accelerates hydrolysis. At this point, pepsin begins the breakdown of the peptide bonds that comprise the protein molecule, resulting in the release of peptides. After reaching the small intestine, trypsin is largely responsible for continuing the digestion of proteins. After gastrointestinal digestion, protein is degraded into oligopeptides, which are further digested into amino acids by the brush-border cells in the intestinal wall, and are subsequently absorbed into the body’s fluid circulation [83,84]. Nonetheless, a portion of nitrogen-containing compounds escape the gastrointestinal tract and reach the large intestine. Researchers have found that nitrogen-containing compounds in the colon serve as nitrogen sources for intestinal microbial fermentation, in addition to contributing to the production of SCFAs. This is also accompanied by the production of toxic metabolites such as ammonia, indole, etc. Therefore, there exists the undesirable intake of certain proteins which may lead to an increased risk of various diseases, such as inflammatory bowel disease and colon cancer [85,86]. Moreover, there are glycated proteins in the daily diet, which are produced by Maillard reactions between proteins and reducing sugars. The low digestibility of these glycated proteins results in more protein entering the distal colon for fermentation [86]. 

Because of its complexity, protein digestion is often simulated by in vitro models, including marine-derived protein. Most of the protein in the ocean comes from fish and algae, among which fish is a high-quality source of collagen. The reserves of these marine proteins are relatively large, so ensuring full use can effectively reduce the waste of resources, which is conducive to ecologically sustainable use. In Yang’s study [87], the in vitro digestion and colonic fermentation behavior of glycosylated fish protein was investigated. Glycosylated fish protein with different heating times (24/48 h) were subjected to in vitro digestion and then a batch fermentation model was applied to simulate colonic fermentation. Fish protein glycosylation was observed to be positively linked to the abundance of *Lactococcus*, especially at 48 h of colonic fermentation. Analysis confirmed that fish protein glycosylation also reduced ammonia and indole production, with potential implications for host health. Han et al. [88] also explored the in vitro digestion and fermentation behavior of glycated myofibrillar protein from grass carp as well as their effects on gut microbiota. Glycosylation reduced myofibrillar protein digestibility as measured by molecular weight. It also enhanced diversity of the fecal microbiota, with potentially positive effects on gut health. Because the digestion and absorption of most proteins finish in the gastrointestinal tract, researchers often use in vitro digestion models to evaluate the digestibility and bioaccessibility of protein and their hydrolysates. According to Karina’s research [89], an in vitro static gastrointestinal digestion model was used to determine the bioaccessibility of protein hydrolysates from *Cynoscion guatucupa*, which exhibited potential bioaccessibility in the gastrointestinal tract, helping to improve bioavailability of some fragments (such as 1500~1000 Da, Phenylalanine and Tryptophan). The proteolysis extent of algae protein was evaluated by a variety of in vitro models. For example, in *Arthrospira platensis* it was about 81%, while in *Phaeodactylum tricornutum* it was only 35% [90,91]. The combination of in vitro gastrointestinal digestion and colonic fermentation models can effectively simulate the complex process of protein digestion and allow for comprehensive evaluation of protein digestion and absorption, which is of great significance.

### 4.3. Lipids

Lipids are the other major nutrient required by human beings, which should provide 35–40% of energy needed by the human body and which participate in metabolism. Dietary lipids can be derived from a variety of animal and plant foods, such as livestock and poultry products, fish, nuts, etc. [92]. Among these dietary lipids, triglycerides (TG) account for about 95%, followed by phospholipids and cholesterol [82]. As in the case of carbohydrates or protein, lipids must be hydrolyzed during digestion in order to be absorbed and exert their biological function. After food is ingested, lipids form blocks with other food components under the action of chewing and intraoral enzymes, and the structure is looser for later digestion. In the stomach, various surfactants are mixed with the chyme, and in the highly acidic environment part of the TG begins to hydrolyze due to the action of gastric lipase, accounting for approximately 10–30% of lipid digestion. However, the small intestine is the main contributor to the digestion of TG. This is due to pancreatic lipase and surface-active components, such as bile salts that interact with the oil to change the interface properties, thus facilitating access by lipases. Thus, oil droplets are mixed with pancreatic lipase and are activated at the oil–water interface, allowing for TG hydrolysis in the free fatty acids and sn-2 monoglycerides [93,94]. The absorption of fatty acids is different depending on molecular weight. The small intestine epithelial cells are mainly responsible for the absorption of small and medium molecular weight fatty acids, while large molecular weight fatty acids enter the lymphatic system and form lipoproteins [95]. Unlike other lipid sources, the ocean is a unique source of polyunsaturated fatty acids (PUFAs), including eicosapentaenoic acid (EPA) and docosahexaenoic acid (DHA), which are widely found in blue fish, algae, and other marine organisms. These PUFAs have been shown to have various health-promoting effects such as anti-inflammatory, prevention of cardiovascular disease or diabetes, and overall showing important implications for host health [96]. Exploring the digestion pathway of marine lipids in the digestive system can better improve the stability and absorption rate of lipids. Dasilva et al. [97] simulated the gastrointestinal digestion of fish oil rich in EPA and DHA with the TIM model, including the stomach, duodenum, jejunum and ileum. Taking into account that the molar ratio of EPA and DHA affects the degree of lipid oxidation and bioaccessibility, the results showed that when the ratio of EPA:DHA was 1:1, the oxidation degree of PUFA was the lowest after simulated digestion, and a high assimilation rate could be maintained. Considering the high absorption rate of TG in the intestinal tract (which can reach 98%), and the solubility of lipids, more researchers choose to use in vitro gastrointestinal digestion models to evaluate the bioaccessibility of lipids, while the potential effects of lipids on gut microbiota are explored through animal models [84]. For example, Francisco et al. [98] established an in vitro digestion model from the oral cavity to the small intestine to study the bioaccessibility of various bioactive compounds from fresh and frozen *Fucus Spiralis*, including fatty acids, antioxidant capacity, etc. It was found that the bioaccessibility of total lipids in *Fucus Spiralis* was about 12%, from which about 7.5% is EPA, and the bioaccessibility of EPA was about 13%, as freeze-dying reduces the bioaccessibility of the bioactive compounds. Because of the special relevance of lipids in infancy, the in vitro digestion in specific models of infants’ digestion have also been used. Canelli et al. [99] assessed bioaccessibility of lipids from *Chlorella vulgaris* using an infant in vitro digestion model, in which the bioaccessibility of lipids was approximately 0.66–2.41%. The digestion and absorption of lipids is an extremely complex process, and lipases and lipid types play an important role in the entire process. As lipids are insoluble in water, they are usually emulsified by surfactant agents into the water environment of the gastrointestinal tract, improving the digestibility and bioaccessibility of fat-soluble components. These marine lipids rich in PUFAs can regulate the gut microbiota and have implications in improving a variety of metabolic diseases. On the other hand, the effect of lipids on the gut microbiota has been more explored through animal models [82].

### 4.4. Polyphenols and Other High-Value Components

From marine organisms, algae are the main source of polyphenols, mainly phloroglucinols and their polymers, phlorotannins. Other polyphenols found in algae are phenolic acids, tichocarpols or bromophenols, which show higher bioactivity than terrestrial plant polyphenols. The phlorotannins of algae present with a wide diversity of structures and a degree of polymerization. In this sense, some of the identified chemical structures of this type of polyphenols include tetrafucol, tetraphlorethol, fucodiphlorethol, tetrafuhalol, tetraisofuhalol and phlorofucofuroeckol. The relevance of algal phlorotannins compared to terrestrial tannins is that the chemical structures include 8 phenolic rings vs. 3–4, conferring a higher antioxidant capacity [100]. Algae is the main source of marine polyphenols, such as phlorotannin, phenolic acids, tichocarpols, etc., which show higher bioactivity than terrestrial plant polyphenols. Marine polyphenols have been proven to have various health benefits, such as antioxidant, anti-tumor and anti-diabetic activities, thus showing great potential for development as functional food ingredients and dietary supplements [101]. Polyphenol structures are bound in the food matrix, and their health properties and effects on the gastrointestinal tract depend on the rate of intestinal release and absorption. The main form of phenolic compounds are polymers, glycosides, etc., so they cannot be digested and absorbed very well in the gastrointestinal tract. However, polyphenols still exhibit high bioactivity even at low absorption rates, so the digestion and metabolism of polyphenols has attracted attention. Both digestive enzymes and gut microbiota play an important role in the digestion of polyphenols. Polyphenols undergo glycoside hydrolysis, methylation, and sulfation reactions in the small intestine. About 45–50% polyphenols are absorbed by the small intestine and enter the body fluid circulation. Undigested polyphenols enter the large intestine, and are further degraded into low molecular weight phenolic acids via the action of the gut microbiota. However, during this process, about 10% of polyphenols remain in the food matrix and cannot be absorbed [102,103]. Understanding the release of polyphenols through in vitro digestion and colonic fermentation models is of great value to improve their bioaccessibility. Vázquez-Rodríguez et al. [75] explored the effect of phlorotannin from *Silvetia compressa* on gut microbiota using in vitro digestion and colonic fermentation models. An oral-to-duodenum digestion model was established to digest ethanolic extracts from *Silvetia compressa*, which was thereafter subjected to in vitro colonic fermentation. The phlorotannin-enriched extracts were found to promote the proliferation of *Bifidobacterium* and *Lactobacillus* species, as well as the synthesis of SCFAs in the colonic fermentation models, with positive effects on gut microbiota. Similarly, Corona and co-workers [104] evaluated the bioactivity of *Ascophyllum nodosum* phlorotannin-rich polyphenol extracts by simulating gastric small intestinal digestion and batch colonic fermentation, and they also evaluated the effect on human colorectal adenocarcinoma cells (HT-29 cells). Although the antioxidant capacity of phlorotannin was significantly reduced after digestion and colonic fermentation, the inhibitory effects on HT-cells growth was enhanced. In addition, the bioaccessibility of polyphenols was also affected by the food matrix, and polyphenols interacted with certain components such as lipids, proteins and carbohydrates. In addition, polyphenol-protein aggregated particles have been shown to increase polyphenol concentration during in vitro digestion and retain higher antioxidant capacity. The addition of dietary lipids increased the bioaccessibility of polyphenols in radishes, with higher bioactivity having been observed. However, there are few reports on marine-derived polyphenols [105,106]. 

In addition to the major nutrients mentioned above, there are also high-value components such as pigments, vitamins, minerals and other structures in marine organisms, and their digestion is also being investigated. Most studies have focused on establishing oral-gastric small intestine digestion models to assess the bioaccessibility of high-value components, aiming to understand their digestive processes and improve absorptivity, but less explored is their effects on colonic fermentation [107,108]. In vitro digestion models can also be used to evaluate the bioaccessibility of other substances in aquaculture, such as drug residues, heavy metals, toxins, etc. Alves et al. [109] evaluated the bioaccessibility of multiple lipophilic and hydrophilic toxins in marine species through an in vitro digestion model. Among them, the bioaccessibility of fat-soluble okadaic acid ranged from 69 to 74%, and the hydrophilic domoic acid, PSP toxins, and total tetrodotoxin showed the ability of absorption by human intestinal epithelial cells. In addition, steaming reduced the bioaccessibility of total tetrodotoxin (from 100% raw to 59% steam). In turn, Cruz et al. [110] explored the bioaccessibility of endocrine disruptors polybrominated diphenyl ethers (PBDEs) and their methoxylated metabolites (MeO-PBDEs) in cooked seafood (cod and salmon) using a multi-compartment in vitro digestion model.

The results showed that the bioaccessibility of these two substances in the small intestine was less than 24%, and cooking could effectively reduce the amount of these contaminants in fish. Furthermore, Fu et al. [111] used the SHIME model to investigate the digestion of the heavy metal Arsenic (As) in a variety of raw and cooked seafood (11 fishes, 10 shellfishes, and 3 others) and its effect on the gut microbiota. These authors observed that boiling reduced As in fish and shellfish by 22.24% and 32.27%, respectively. They also found the digestibility of As in different seafood was: gastric stage (fish 68.6% > shellfish 40.9% > algae 31%) and intestinal stage (fish 81.9% > algae 53.6% > shellfish 52.5%), while there was no obvious difference in colonic stage. This study helps to reveal the changes of As during digestion under the influence of digestive juice and gut microbiota. Therefore, in vitro models can also be used to evaluate the potential risks that harmful substances may pose to human health. The overall findings regarding the effect of simulated digestion of marine compounds are summarized in Table 3.

## 5. Conclusions and Future Perspectives

Marine organisms are rich in a variety of high added value compounds needed by humans. At present, some high value nutrients from marine organisms are used in the food industry to improve food quality, enhance nutritional value, etc. Meanwhile, some compounds have antioxidant, anti-inflammatory, and anti-coagulation bioactivities, which make them useful for the pharmaceutical industry. The establishment of in vitro digestion models can help people predicting the potential effects of marine bioactive compounds on human health. Through in vitro digestion and colonic fermentation models, their behavior in the complex digestive system can be simulated, where the interaction between the food matrix and the digestive system can be assessed. However, food is often rich in a variety of nutrients, and the digestion of food is affected by multiple factors, including the complexity of the food, particle size, etc. Whether it is a static or a dynamic model, more current studies focus on a single nutrient, which has a certain gap with the actual digestion process. Furthermore, in vitro digestion and fermentation models cannot simulate all systems related to nutrient digestion and absorption, and different reagents, enzyme and inoculum may have different consequences for the results. Therefore, it is necessary to explore more standard and reliable in vitro models. Researchers are exploring complex digestion models that are closer to the real digestion process through various methods, including mathematical models, computer simulations, etc., but it is still a huge challenge. The digestion and absorption of complex foods rich in marine bioactive compounds remains one of the future research topics. In the context of a booming population and increasing demand for healthy foods, it is aimed at providing a basis for the development of rational diets or foods with special digestive properties.

## Figures and Tables

**Table 1:** Advantages and disadvantages of in vitro digestion model.

Model	Stage	Advantage	Disadvantage
INFOGEST	Oral-gastric-small intestine	Simple operation, short time consuming, suitable for single component digestion. Several samples simultaneously	The physiological environment is simplified and cannot simulate dynamic processes (digestive fluids secretion flow rate, removal of the products of digestion).
DGM	Gastric (Fundus/main body and antrum)	Digestion and emptying of food in the stomach can be monitored in real-time.	The position of the main body and the gastric antrum is different from the real; it is necessary to combine the duodenum model to track the further form changes of food after DGM
HGS	Gastric (Antrum)	The mechanical force is more reasonable, and the digestion parameters can be changed.	Only simulates stomach digestion, with limitations; compartments fail to model the true shape of the stomach.
DIDGI	Gastric-small intestine	The device is transparent and the morphological changes of food during digestion can be monitored.	Absorption in the small intestine phase has not been simulated and needs to be combined with other models.
TIM	Gastric and small intestine (duodenum, jejunum and ileum)	Simulates the complete digestive system and can be used to explore the digestive process of various foods.	Inaccurate simulation on shear/grinding of the gastrointestinal tract.
SHIME	Oral-gastric-small and large intestine	Simultaneous sampling and automatic parameter definition: adding fluid flow rate, emptying time and flow rate, with better stability and reproducibility.	Experiments take at least 4–5 weeks and the equipment is fed 3 times a day with the study compounds for at least 2 weeks. More suitable for studies of extracts or pure substances. In addition, the absorption of metabolites was not considered.
ESIN	Oral, stomach, duodenum, jejunum and ileum	Ability to simulate digestion of foods of a similar size to normal meals.	Simulates digestion from oral to small intestine only, needs to be combined with colonic fermentation models.
SIMGI	Stomach, small intestine, colon	Digestion parameters can be controlled, including digester flow rate, digestion volume and time, pH, temperature and pressure, etc.	Absorption of metabolites and interactions between gut microbiota and host cannot be simulated.
ARCOL	Colon	The anaerobic environment of the colon and the passive absorption of metabolites can be simulated.	The different physiological conditions of the three parts of the colon cannot be distinguished.

**Table 3:** Effect of digestion on marine compounds.

Bioactive Compounds	Oral	Gastric	Small Intestinal	Colon
Polysaccharides	It is not degraded by digestive enzymes			Participate in the fermentation of gut microbiota and increase the abundance of beneficial bacteria such as *Bifidobacterium* and *Lactobacillus*.
Protein		Structural degeneration	It is degraded into oligopeptides and amino acids, which enter the body fluid circulation.	The undigested protein enters the distal colon and supplies nitrogen to the gut microbiota.
Lipids	The structure starts to fall apart	Partial lipid hydrolysis	The lipids are hydrolyzed into smaller molecules of fatty acids that are absorbed by the intestine.	
Phenolic	Digestive enzymes hydrolyze food matrix and release polyphenols		Polyphenols undergo glycosidic hydrolysis and methylation, and some of them are absorbed by small intestinal.	Undigested polyphenols are degraded into phenolic acids, which participate in colonic fermentation.

## Data Availability

Not applicable.
